# Supplementary material for: Pectobacterium versatile Bacteriophage Possum: A Complex Polysaccharide-Deacetylating Tail Fiber as a Tool for Host Recognition in Pectobacterial Schitoviridae
Source: Int J Mol Sci. 2022 Sep 20;23(19):11043. doi: 10.3390/ijms231911043 (PMC9569702; doi:10.3390/ijms231911043)
Supplement: Supplementary file 1 [file ijms-23-11043-s001.zip › ijms-1902998-supplementary.pdf]

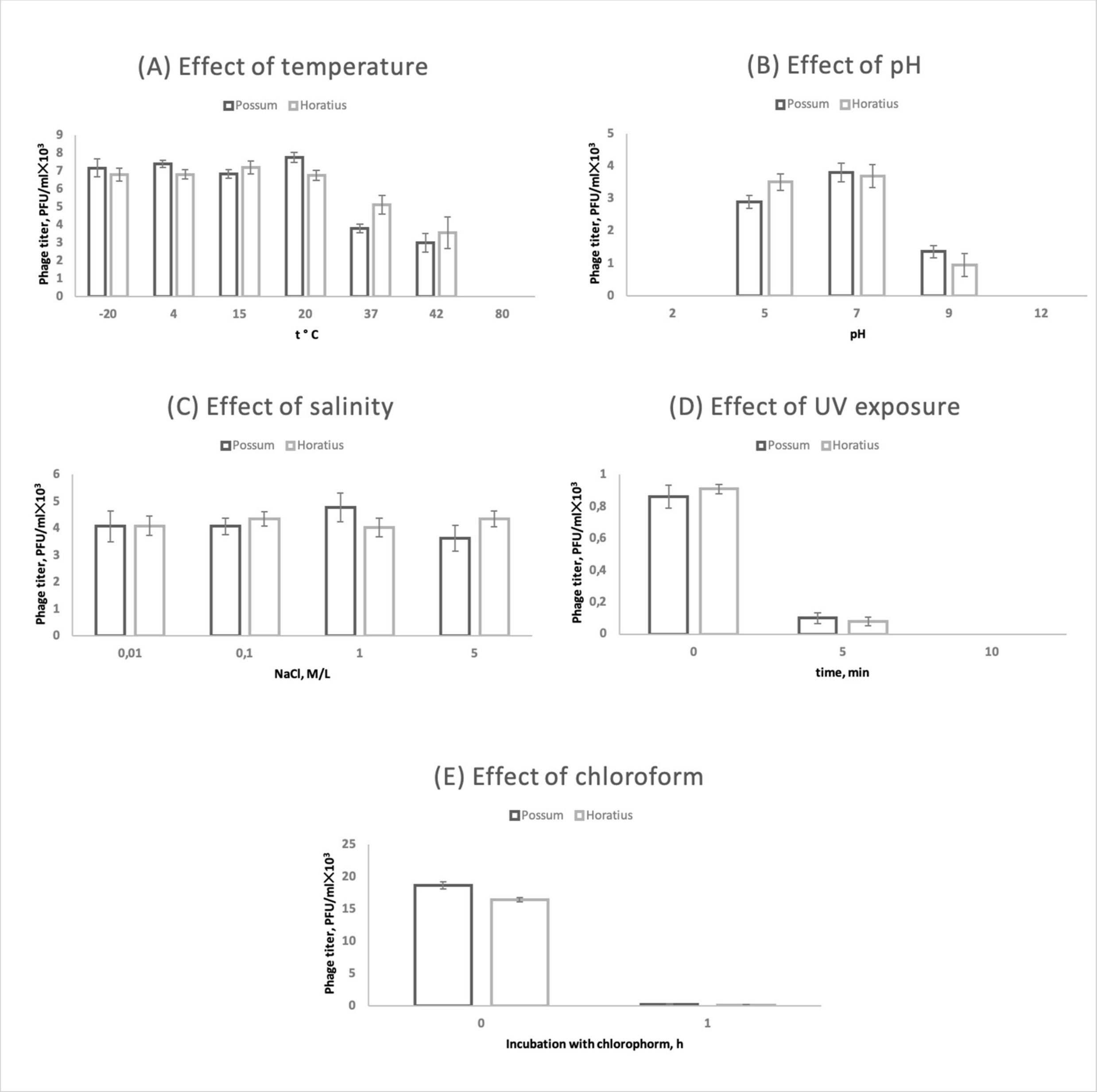

Resistance of bacteriophages Possum and Horatius to environmental factors - temperature, pH, salinity, ultraviolet radiation and chloroform treatment

Figure S1. Resistance of bacteriophages Possum and Horatius to environmental factors: temperature, pH, salinity, ultraviolet radiation, and chloroform treatment.

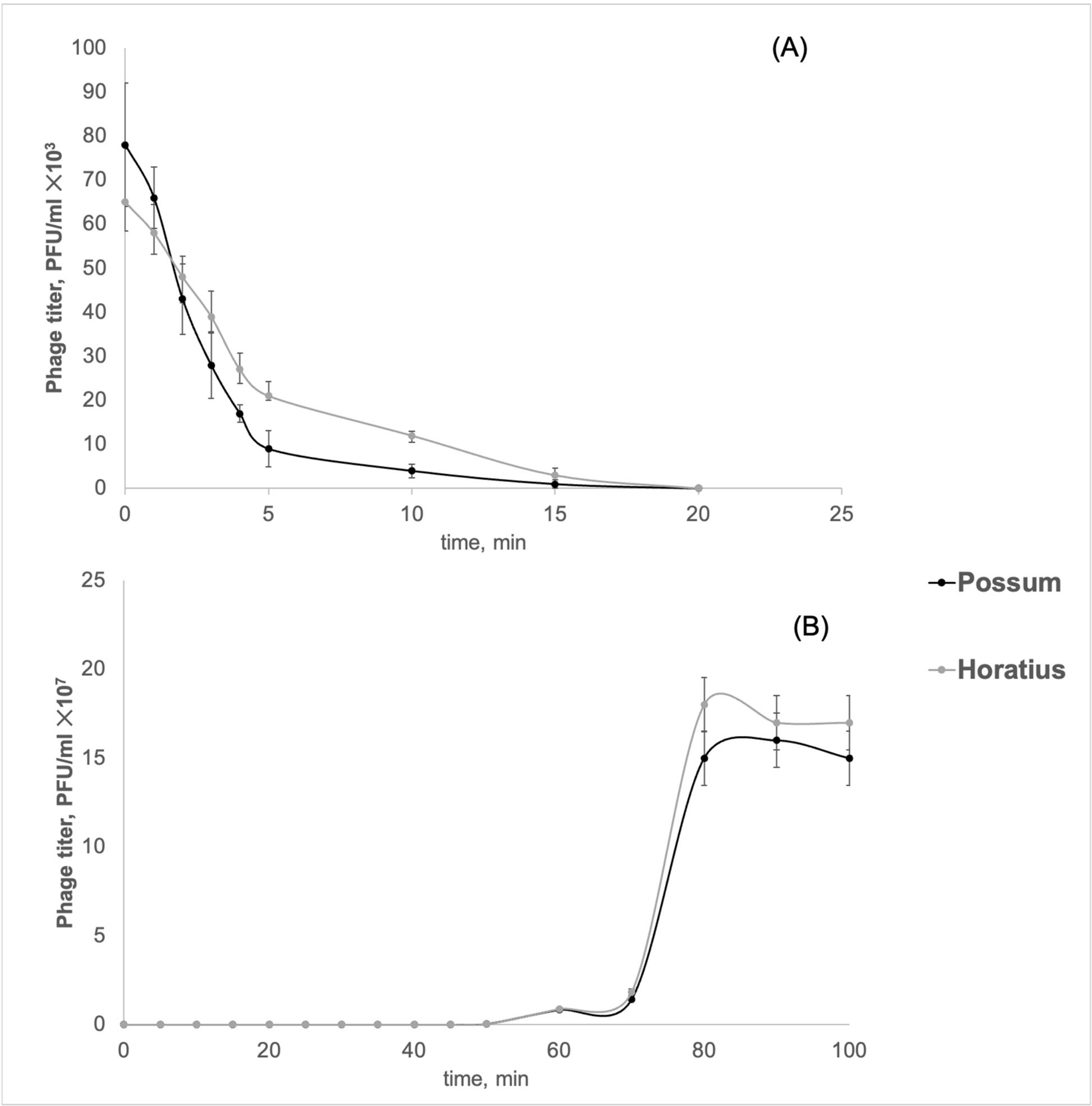

Adsorption curve (top) and One Step Growth (bottom) of bacteriophages
Possum and Horatius using P. versatile F131 isolation host at 28°C with MOI = 0.001 and 0.01 for adsorption and OSG, respectively

Figure S2. Adsorption curve (A) and one-step growth (B) of bacteriophages Possum and Horatius using *P. versatile* F131 isolation host at 28 °C with MOI = 0.001 and 0.01 for adsorption and OSG, respectively.

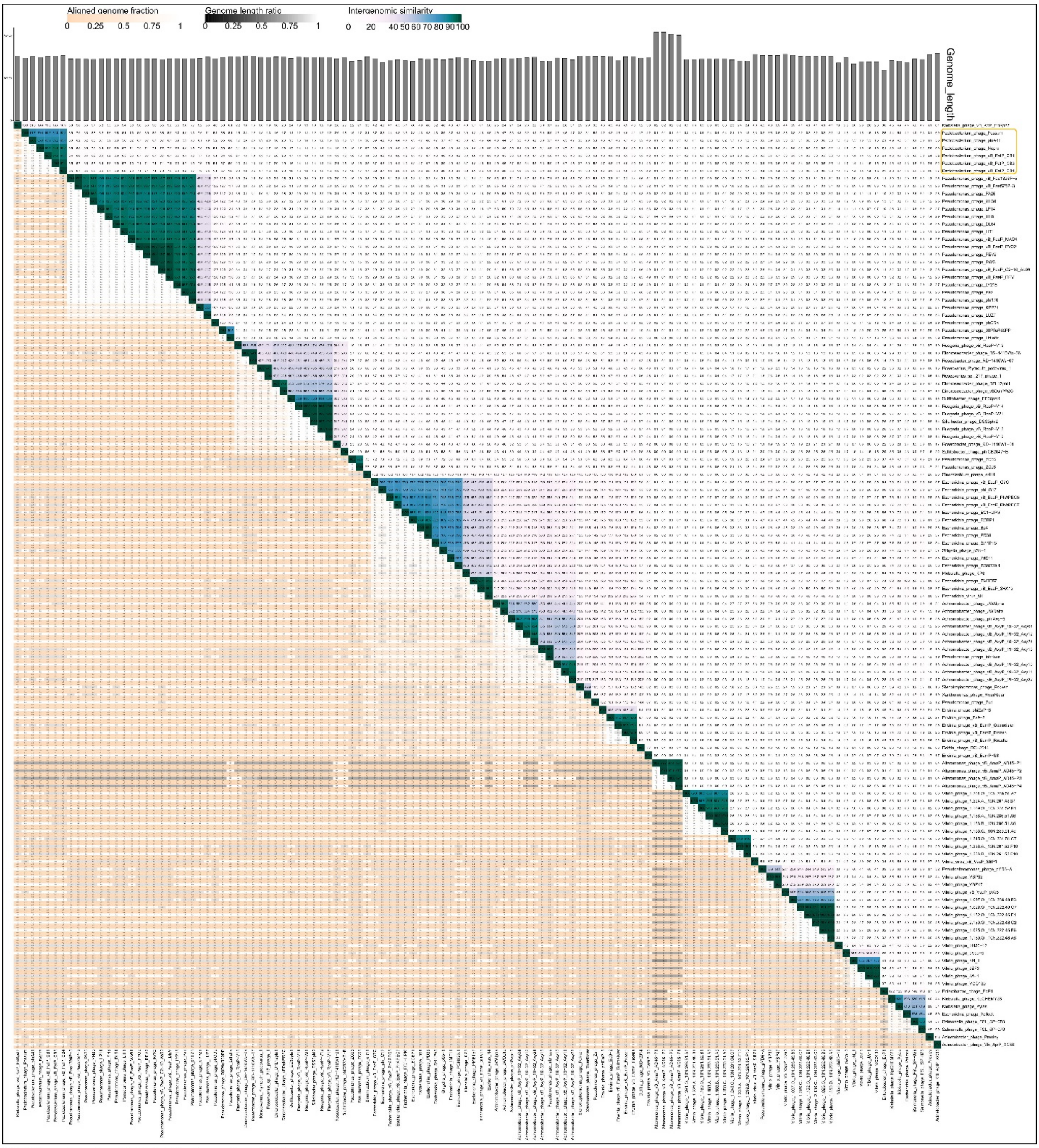

VIRIDIC generated heatmap of 118 *Schitoviridae* phages and four related *Aeromonas* phages.
The heatmap incorporates intergenomic similarity values (right half) and alignment indicators (left half and top annotation). In the right half, the colour coding indicates the clustering of the phage genomes based on intergenomic similarity. The numbers represent the similarity values for each genome pair, rounded to the first decimal. In the left half, three indicator values are represented for each genome pair, from top to bottom: aligned fraction genome 1 (for the genome found in this row), genome length ratio (for the two genomes in this pair) and aligned fraction genome 2 (for the genome found in this column). Pectobacterium phages are clustered with an intergenomic similarity higher than the genus threshold of 70%

Figure S3. VIRIDIC generated heatmap of 118 *Schitoviridae* phages and four related *Aeromonas* phages. The heatmap incorporates intergenomic similarity values (right half) and alignment indicators (left half and top annotation). In the right half, the color coding indicates the clustering of the phage genomes based on intergenomic similarity. The numbers represent the similarity values for each genome pair, rounded to the first decimal. In the left half, three indicator values are represented for each genome pair, from top to bottom: aligned fraction genome 1 (for the genome found in this row), genome length ratio (for the two genomes in this pair), and aligned fraction genome 2 (for the genome found in this column). *Pectobacterium* phages are clustered with an intergenomic similarity higher than the genus threshold of 70%.

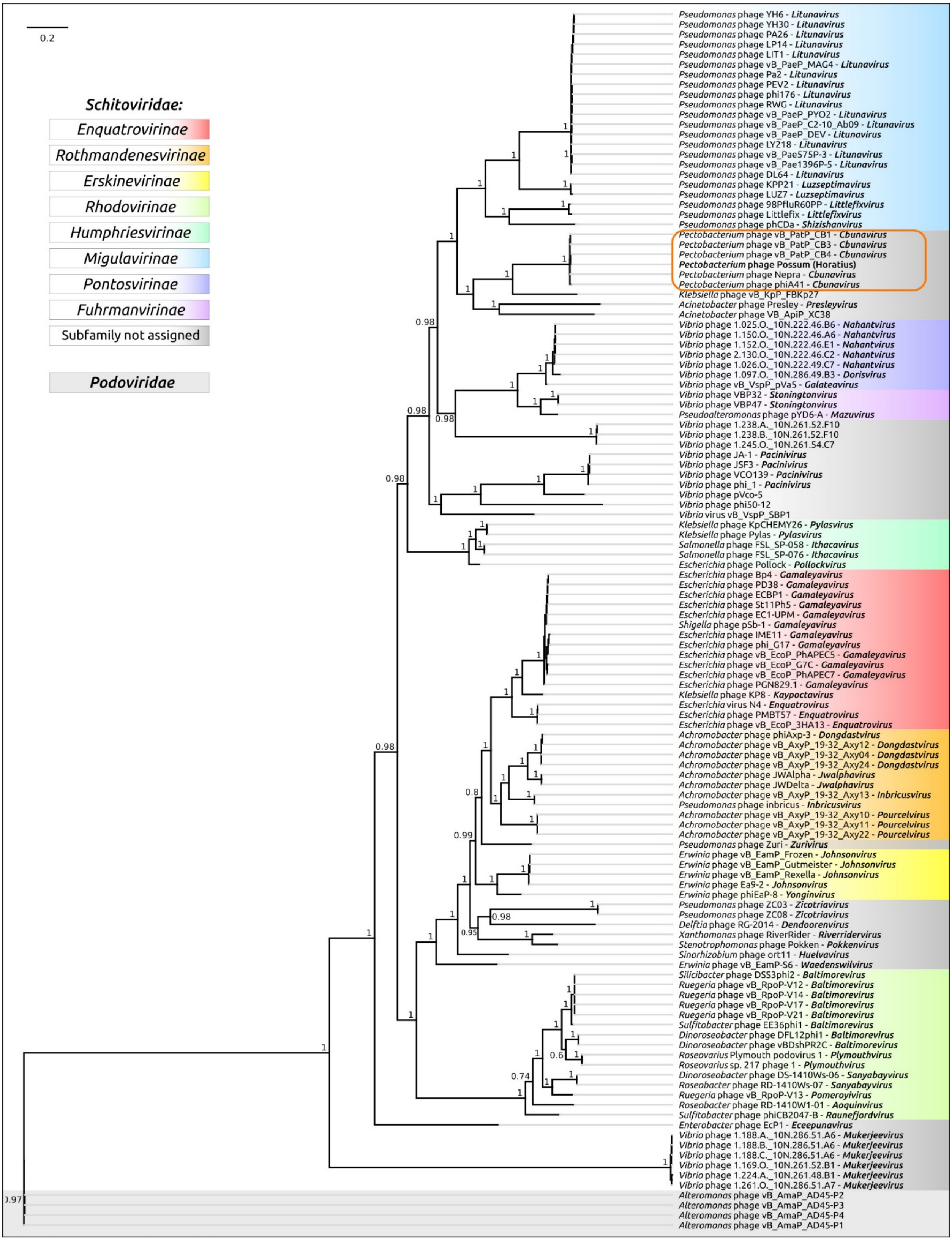

Figure S4. Phylogenetic tree obtained with MrBayes, based on concatenated amino acid sequences of major capsid protein, portal protein, and large subunit of terminase. Bayesian posterior probabilities are indicated above their branch. The scale bar shows 0.2 estimated substitutions per site, and the tree was rooted to representing *Alteromonas* phages vB_AmaP_AD45-P1; 2,000,000 generations sampled every 200 generations, with an average standard deviation of split frequencies of 0.0065.

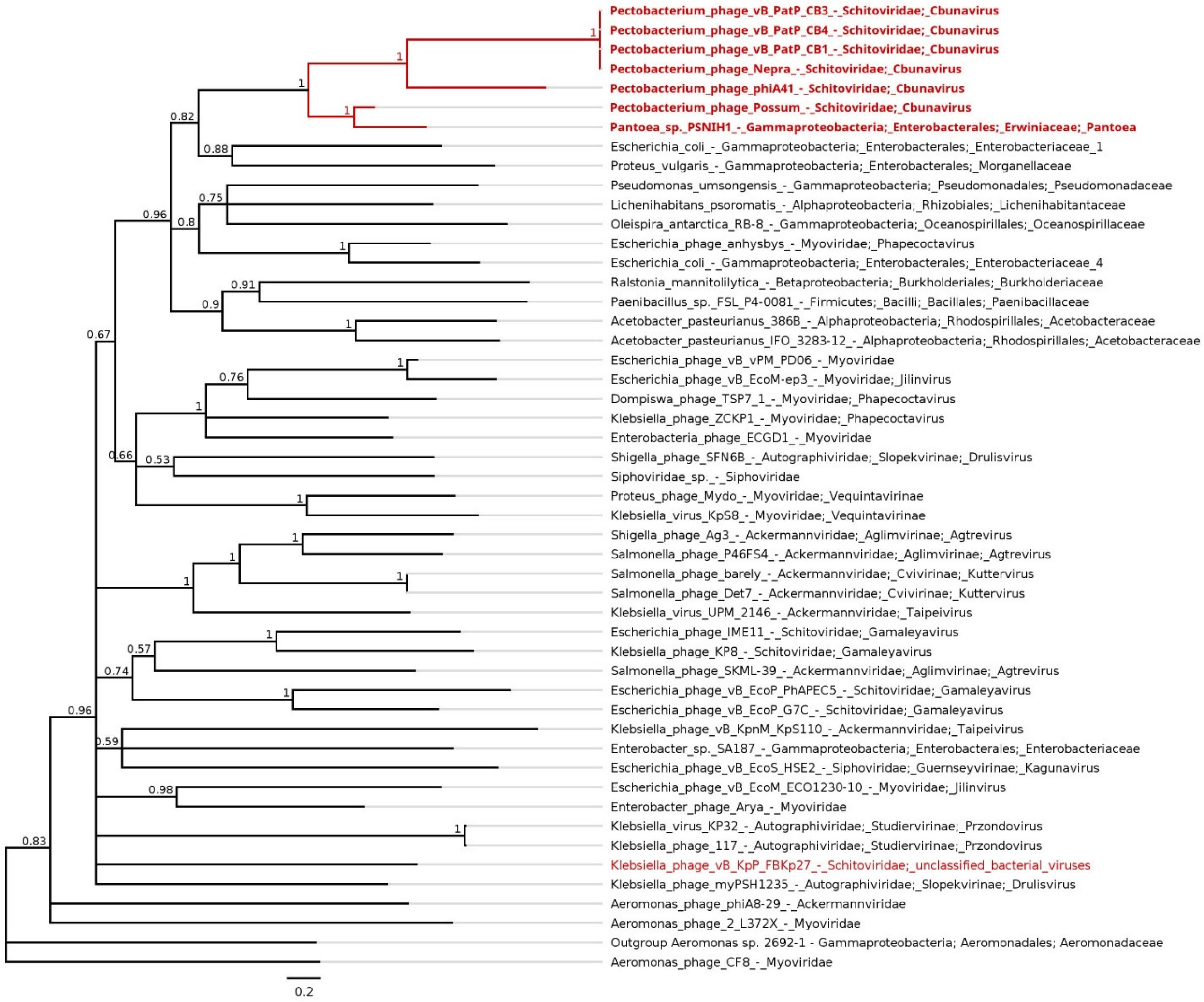

Phylogenetic tree obtained with MrBayes, based on the amino acid sequences homologues to Possum tail fibre protein gp66. Bayesian posterior probabilities are indicated above their branch. The scale bar shows 0.2 estimated substitutions per site and the tree was rooted to representing *Alteromonas* phages vB_AmaP_AD45-P1; 4,000,000 generations sampled every 200 generations, with an average standard deviation of split frequencies of 0.027;

Figure S5. Phylogenetic tree obtained with MrBayes, based on the amino acid sequences homologues to Possum tail fiber protein gp66. Bayesian posterior probabilities are indicated above their branch. The scale bar shows 0.2 estimated substitutions per site, and the tree was rooted to representing *Alteromonas* phages vB_AmaP_AD45-P1; 4,000,000 generations sampled every 200 generations, with an average standard deviation of split frequencies of 0.027.

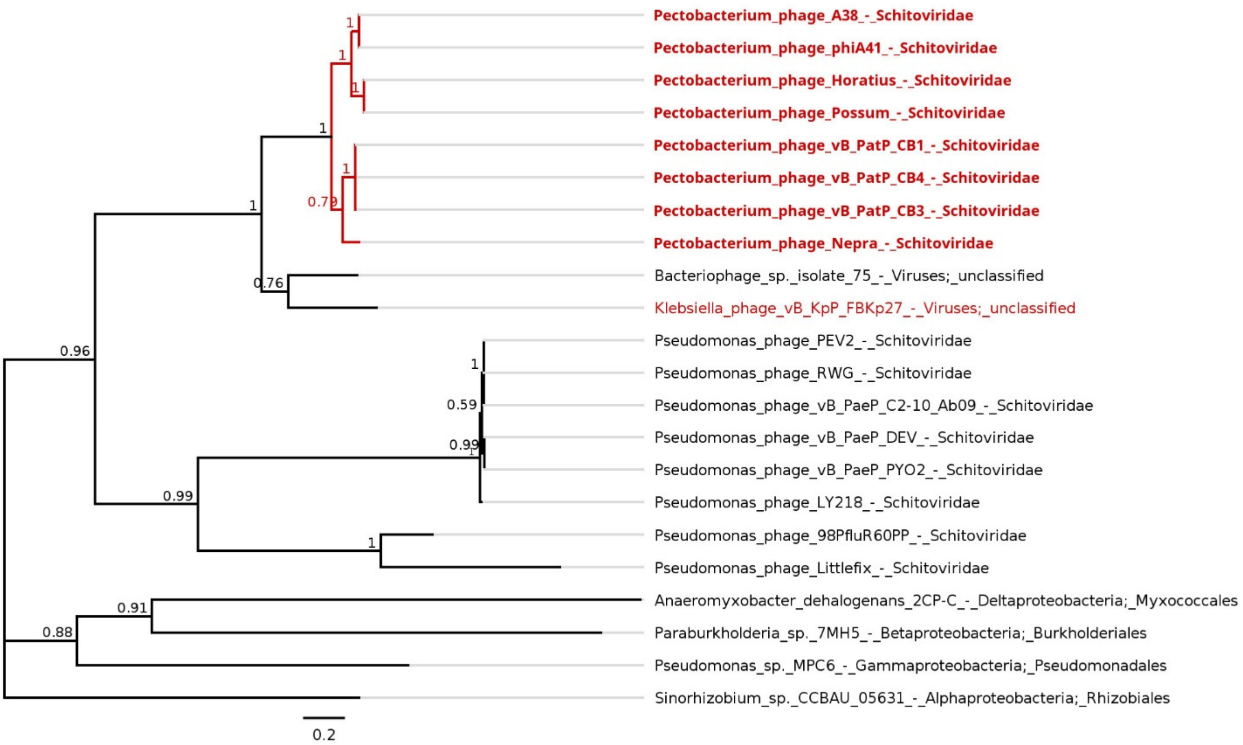

Phylogenetic tree obtained with MrBayes, based on the amino acid sequences homologues to Possum tail fibre protein gp67. Bayesian posterior probabilities are indicated above their branch. The scale bar shows 0.2 estimated substitutions per site and the tree was rooted to representing *Sinorhizobium* sp. CCBAU_05631; 2,000,000 generations sampled every 200 generations, with an average standard deviation of split frequencies of 0.0033;

Figure S6. Phylogenetic tree obtained with MrBayes, based on the amino acid sequences homologues to Possum tail fiber protein gp67. Bayesian posterior probabilities are indicated above their branch. The scale bar shows 0.2 estimated substitutions per site, and the tree was rooted to represent *Sinorhizobium* sp. CCBAU_05631; 2,000,000 generations sampled every 200 generations, with an average standard deviation of split frequencies of 0.0033.

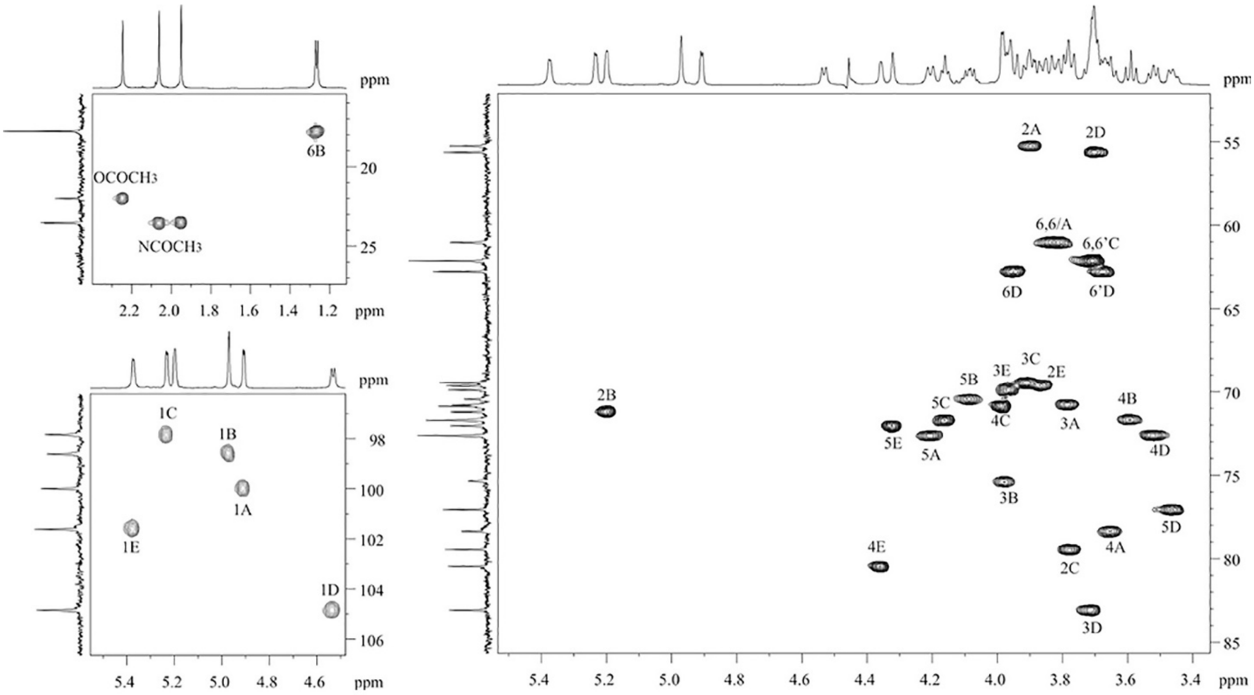

Parts of a 1H,13C HSQC spectrum of the OPS. Arabic numbers refer to carbons in sugar residues denoted by letters as shown in Figure 6 and Table S6

Figure S7. Parts of a 1H,13C HSQC spectrum of the OPS. Arabic numbers refer to carbons in sugar residues denoted by letters as shown in Figure 6 and Table S6.

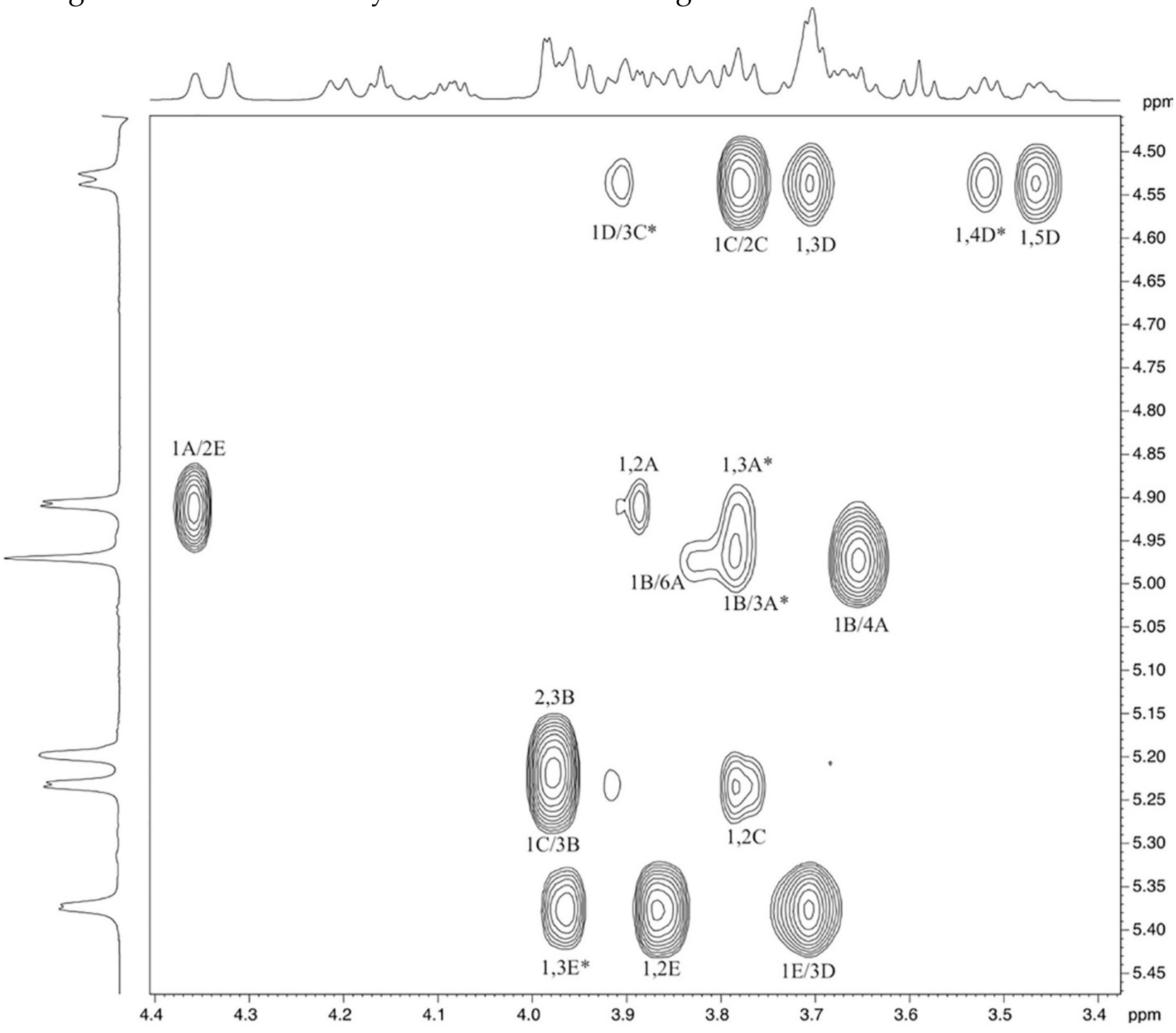

Part of a 1H,1H ROESY spectrum of the OPS. Arabic numbers refer to protons in sugar residues denoted by letters as shown in Figure 6 and Table S6

Figure S8. Part of a 1H,1H ROESY spectrum of the OPS. Arabic numbers refer to protons in sugar residues denoted by letters as shown in Figure 6 and Table S6.

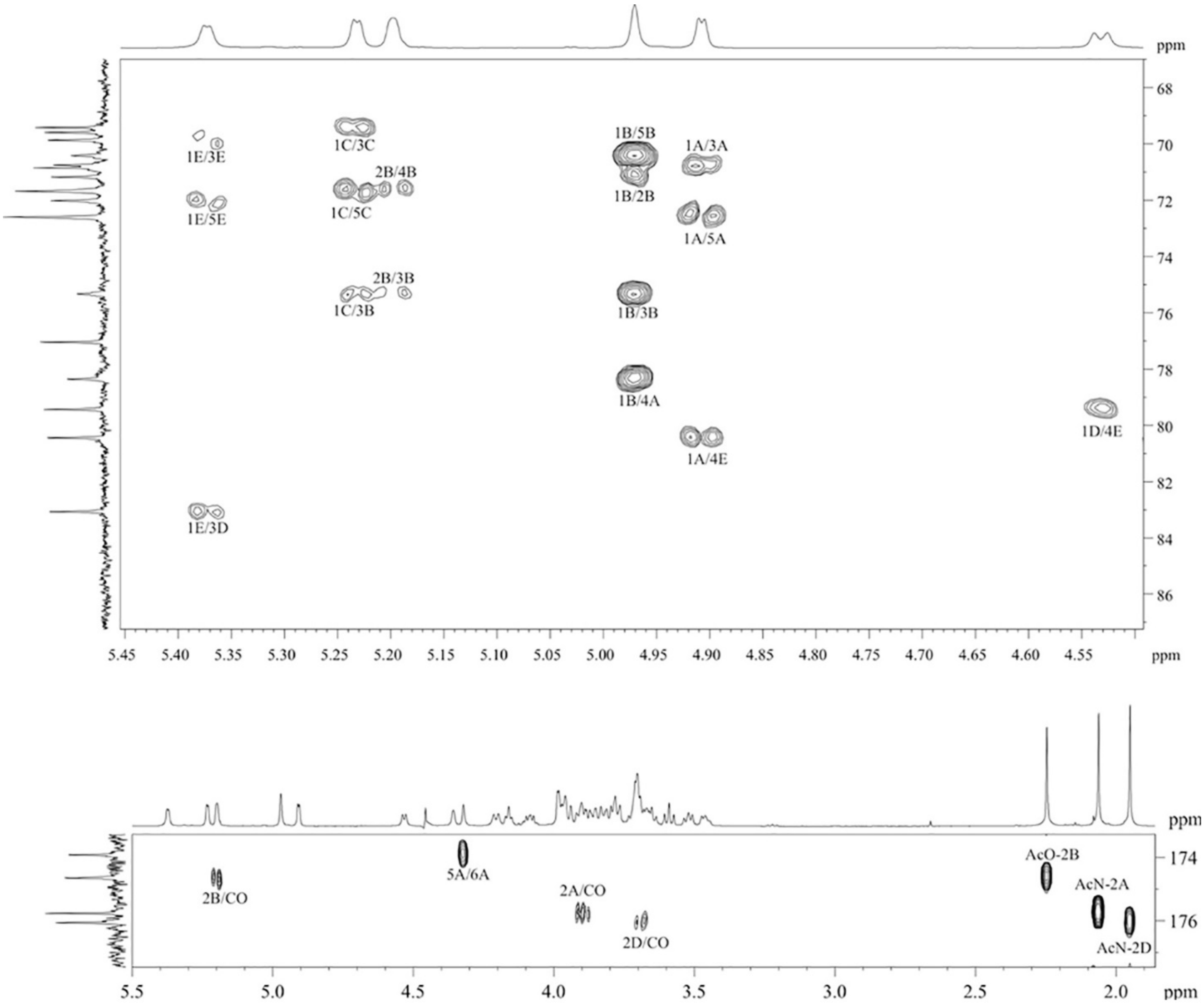

Parts of a 1H,13C HMBC spectrum of the OPS. Arabic numbers before slash refer to protons and those after slash to carbons in sugar residues denoted by letters as shown in Figure 6 and Table S6

Figure S9. Parts of a 1H,13C HMBC spectrum of the OPS. Arabic numbers before slash refer to protons and those after slash to carbons in sugar residues denoted by letters as shown in Figure 6 and Table S6.

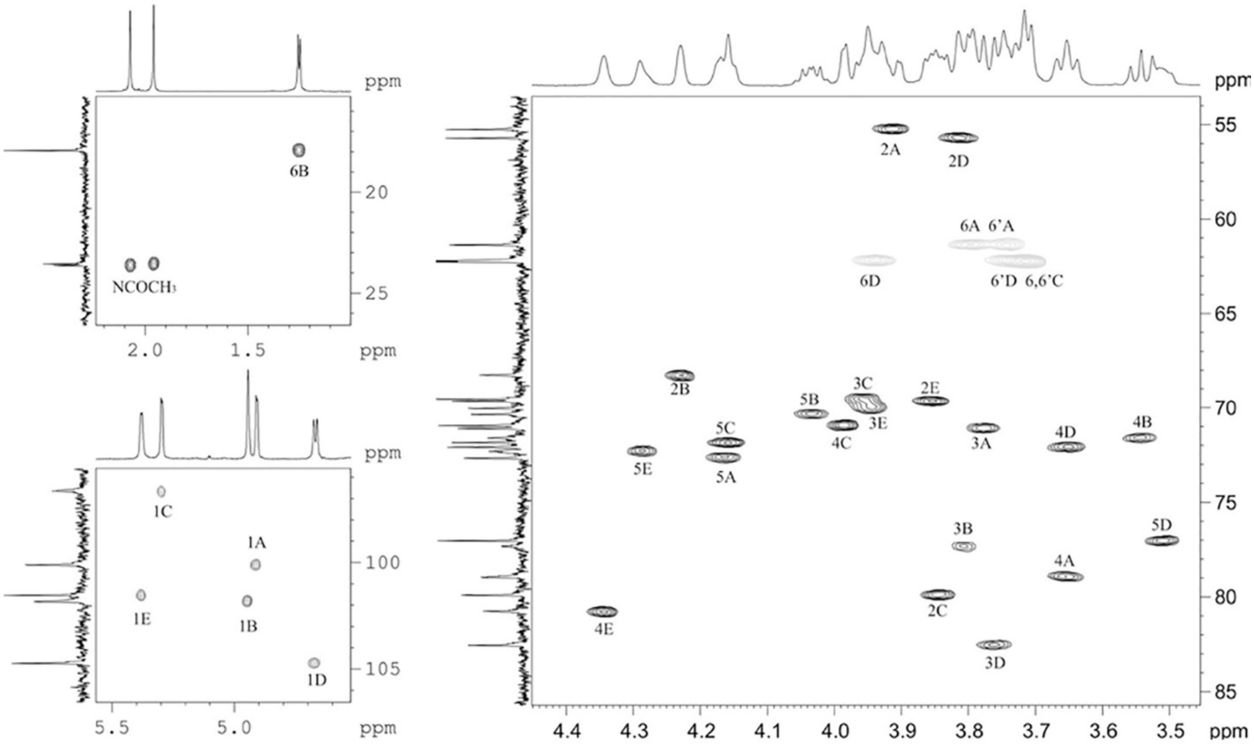

Parts of a 1H,13C HSQC spectrum of the MPS. Arabic numbers refer to carbons in sugar residues denoted by letters as shown in Figure 6 and Table S6

Figure S10. Parts of a 1H,13C HSQC spectrum of the MPS. Arabic numbers refer to carbons in sugar residues denoted by letters as shown in Figure 6 and Table S6.

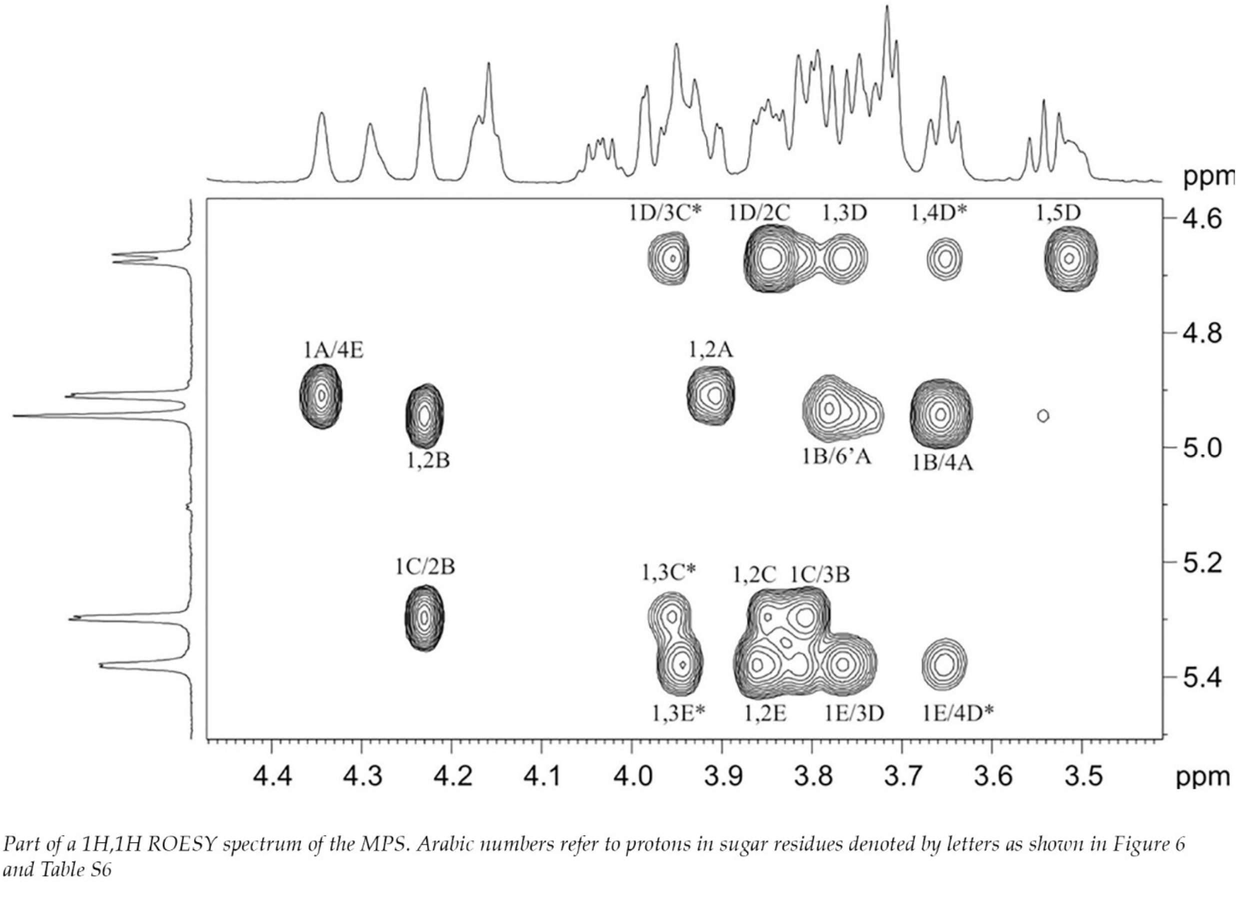

Part of a 1H,1H ROESY spectrum of the MPS. Arabic numbers refer to protons in sugar residues denoted by letters as shown in Figure 6 and Table S6

Figure S11. Part of a 1H,1H ROESY spectrum of the MPS. Arabic numbers refer to protons in sugar residues denoted by letters as shown in Figure 6 and Table S6.

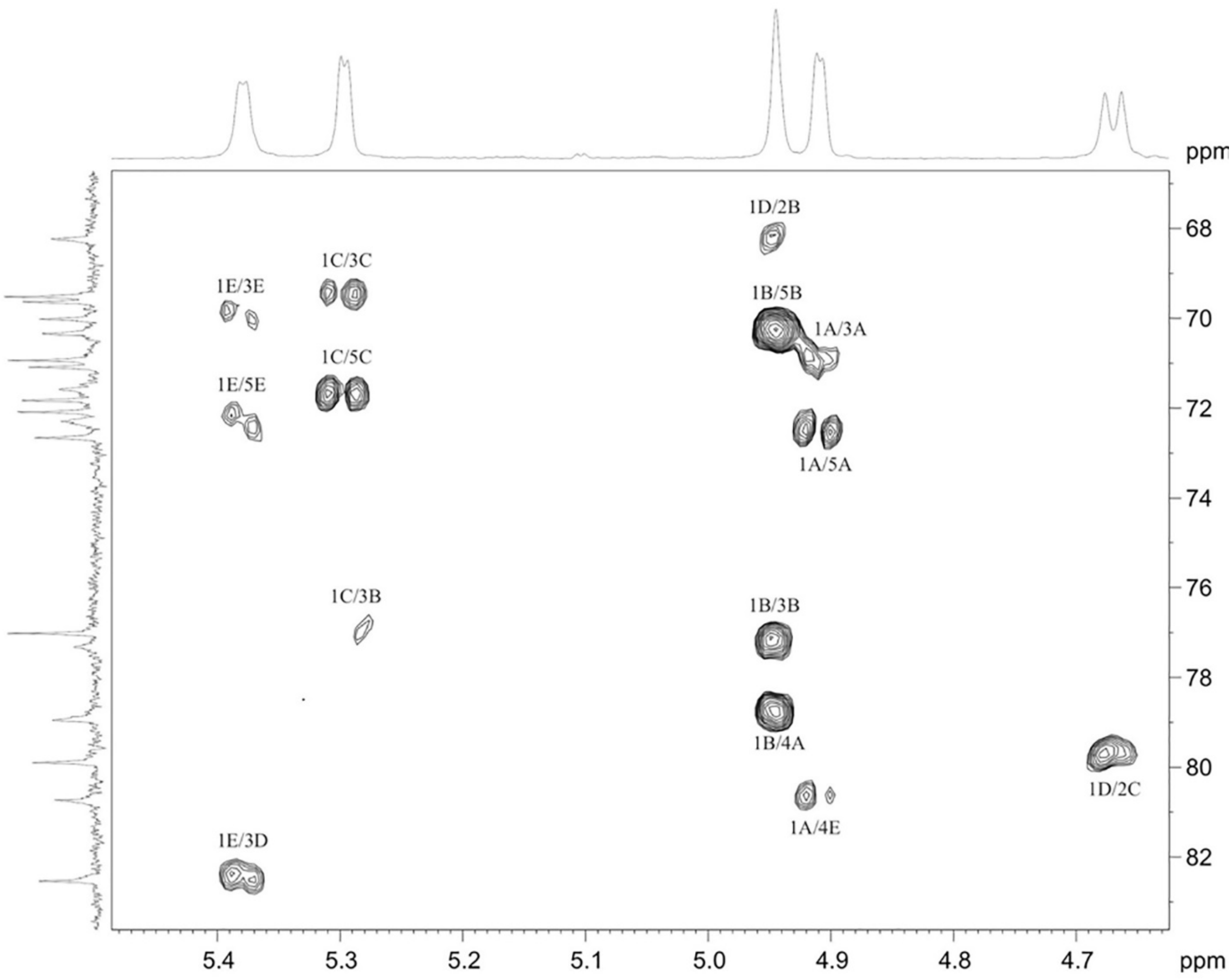

Part of a 1H,13C HMBC spectrum of the MPS. Arabic numbers before slash refer to protons and those after slash to carbons in sugar residues denoted by letters as shown in Figure 6 and Table S6

Figure S12. Part of a 1H,13C HMBC spectrum of the MPS. Arabic numbers before slash refer to protons, and those after slash to carbons in sugar residues denoted by letters as shown in Figure 6 and Table S6.

Table S1. Host Range of bacteriophages Possum and Horatius.

№	Strain	Alternative name	Species	Origin	Genome	Possum	Horatius
**1**	F002	PB69	*Pve*	Moscow Region	NZ_PDVY00000000.1	-	-
**2**	F016		*Pve*	Moscow Region		-	-
**3**	F018		*Pve*	Moscow Region		-	-
**4**	F020		*Pve*	Moscow Region		-	-
**5**	F021		*Pve*	Moscow Region		-	-
**6**	F040		*Pve*	Moscow Region		-	-
**7**	F131	12a	*Pve*	VIZR	NZ_PDVW00000000.1	+	+

**8**	F135		*Pve*	Moscow Region	NZ_PDVX00000000.1	-	-
**9**	F015	D6	*Pca/Pve*	Moscow		-	-
**10**	F017	246 / I7	*Pca/Pve*	Moscow		-	-
**11**	F022	2.4 / F1	*Pca/Pve*	Tomsk		-	-
**12**	F024	A7	*Pca/Pve*	Lithuania		-	-
**13**	F025	B9	*Pca/Pve*	Moscow		-	-
**14**	F027	D1	*Pca/Pve*	Moscow		-	-
**15**	F047	10b	*Pca/Pve*	Moscow		-	-
**16**	F050	10a	*Pca/Pve*	Moscow		-	-
**17**	F061	35 / F8	*Pca/Pve*	Ethiopia		-	-
**18**	F062	246 / I8	*Pca/Pve*	Moscow		-	-
**19**	F063	D3	*Pca/Pve*	Unknown		-	-
**20**	F064	520 / F6	*Pca/Pve*	Moscow		-	-
**21**	F073	5	*Pca/Pve*	Unknown		-	-
**22**	F077	B6	*Pca/Pve*	Ethiopia		-	-
**23**	F092	E2	*Pca/Pve*	Moscow		-	-
**24**	F099	518 / E8	*Pca/Pve*	Moscow		-	-
**25**	F111	301 / I8	*Pca/Pve*	Moscow		-	-
**26**	F121	C8	*Pca/Pve*	Moscow		-	-
**27**	F124	2019.14	*Pca/Pve*	Tver		-	-
**28**	F133	C1	*Pca/Pve*	Tyumen		-	-
**29**	F136		*Pca/Pve*	Bryansk		-	-
**30**	F061		*Pca*	Ethiopia		-	-
**31**	F160		*Pca*	VKPM	NZ_JQHJ00000000.1	-	-
**32**	F164	PB31	*Paq*	Moscow Region		-	-
**33**	F004	PB72	*Pat*	Moscow Region	NZ_PDDK00000000.1	-	-
**34**	F041		*Pat*	Moscow Region		-	-
**35**	F048		*Pat*	Moscow Region		-	-
**36**	F162	SCRI1043	*Pat*	Scotland	NC_004547	-	-
**37**	F163	21A	*Pat*	Belarus	NZ_CP009125	-	-
**38**	F126		*Pbr*	Samara Region	RRYQ00000000	-	-
**39**	F152		*Pbr*	Moscow Region	PJDM00000000	-	-

**40**	F157		*Pbr*	Moscow Region	PJDL00000000	-	-
**41**	F035		*Ppar*	Kaluga Region		-	-
**42**	F148	PB20	*Ppar*	Moscow Region	NZ_PDDJ00000000.1	-	-
**43**	F149	PB21	*Ppar*	Tver region		-	-
**44**	F174		*Ppar*	Tver region		-	-
**45**	F109		*Ppol*	Ethiopia		-	-
**46**	F056		*Ddi*	Moscow Region		-	-
**47**	F085		*Ddi*	Moscow Region		-	-
**48**	F127		*Ddi*	Kaluga Region		-	-
**49**	F012		*Dso*	Voronezh Region	NZ_PGOJ00000.1	-	-
**50**	F155	PB36	*Dso*	Moscow Region		-	-
**51**	F100		*unidentified pectolytic isolate*	Unknown		-	-
**52**	F106		*unidentified pectolytic isolate*	Unknown		-	-

"+" Display phage lytic activity concerning bacterial strains. For strains resistant to phages, the "-" sign is indicated. *Pve – Pectobacterium versatile, Pve/Pca* - strains previously attributed as *Pca,* having a PCR fingerprint similar to *Pve, Pca – P.carotovorum, Pat – P. atrosepticum, Pbr – P. brasiliense, Ppar – P. parmentieri, Ppol – P. Polaris, Ddi – Dickeya dianticola, Dso – D. solani.*

Table S2. Functional assignments of *Pectobacterium* phage Possum genes.
Table S3. Functional assignments of *Pectobacterium* phage Horatius genes.
Table S4. Unique genes belonging to *Pectobacterium* N4-like phages and *Klebsiella* phage vB_KpP_FBKp27 found with BLAST search (E-value <10-5).
Table S5. Average nucleotide identity (ANI) between *Pectobacterium* phage Possum and all phage genomes deposited in the NCBI GenBank (calculated with orthoANIu, threshold 0.5).

Table S6. ^{1}H and ^{13}C NMR chemical shifts (δ, ppm) of the O-specific polysaccharide (OPS) and modified (O-deacetylated) polysaccharide (MPS) from *P. versatile* F131.

Sugar residue	Chemical shift					
	C-1<br>*H-1*	C-2<br>*H-2*	C-3<br>*H-3*	C-4<br>*H-4*	C-5<br>*H-5*	C-6<br>*H-6,6′*
OPS[a]						
→4)-α-D-Glc*p*NAc-(1→ **A**	100.0	55.2[c]	70.8	78.3	72.6	61.0
	4.91	*3.89*	*3.79*	*3.66*	*4.21*	*3.81, 3.84*
→3)-α-L-Rha*p*2Ac-(1→ **B**	98.6	71.1[b]	75.4	71.7	70.5	17.8
	4.97	*5.20*	*3.99*	*3.59*	*4.09*	*1.27*
→2)-α-D-Gal*p*-(1→ **C**	97.8	79.5	69.5	70.9	71.7	62.1
	5.24	*3.78*	*3.91*	*3.99*	*4.17*	*3.71, 3.71*
→3)-β-D-Glc*p*NAc-(1→ **D**	104.9	55.6[a]	83.1	72.6	77.1	62.8
	4.53	*3.70*	*3.72*	*3.52*	*3.47*	*3.68, 3.95*
→4)-α-D-Gal*p*A-(1→ **E**	101.6	69.6	69.9	80.5	72.1	173.9
	5.37	*3.86*	*3.97*	*4.36*	*4.32*	
MPS[b]						
→4)-α-D-Glc*p*NAc-(1→ **A**	100.2	55.3[b]	71.1	79.0	72.7	61.4
	4.91	*3.92*	*3.78*	*3.65*	*4.17*	*3.75, 3.80*
→3)-α-L-Rha*p*-(1→ **B**	101.8	68.3	77.4	71.6	70.3	18.0
	4.95	*4.23*	*3.81*	*3.54*	*4.04*	*1.25*
→2)-α-D-Gal*p*-(1→ **C**	96.7	79.9	69.6	71.0	71.9	62.3
	5.30	*3.85*	*3.96*	*3.99*	*4.16*	*3.71, 3.71*
→3)-β-D-Glc*p*NAc-(1→ **D**	104.7	55.7[a]	82.6	72.1	77.0	62.7
	4.67	*3.82*	*3.76*	*3.65*	*3.52*	*3.75, 3.94*
→4)-α-D-Gal*p*A-(1→ **E**	101.6	69.2	70.0	80.7	72.3	174.6
	5.38	*3.86*	*3.95*	*4.35*	*4.29*	

^{1}H NMR chemical shifts are italicized.

Signals for the N-acetyl groups are at [a]δ_C 23.6 -23.7 (CH_3) and 175.9-176.2 (C=O), δ_H 1.96 and 2.07 (CH_3); [b]δ_C 23.5-23.6 (CH_3) and 175.9-176.1 (C=O), and δ_H 1.95 and 2.06 (CH_3); the O-acetyl group at δ_C 22.0 (CH_3) and 174.7 (C=O), and δ_H 2.25 (CH_3).
